# Bio‐Inspired SA‐FA Bionic Dual Receptor Electronic Skin for Intelligent Gesture and Material Cognition Systems Enhanced by Static‐Dynamic Mutual Interaction

**DOI:** 10.1002/advs.202509740

**Published:** 2025-08-13

**Authors:** Hao Li, Hongsen Niu, Hao Kan, Eun‐Seong Kim, Nam‐Young Kim, Yang Li

**Affiliations:** ^1^ School of Integrated Circuits Shandong University Jinan 250101 China; ^2^ Shandong Provincial Key Laboratory of Ubiquitous Intelligent Computing School of Information Science and Engineering University of Jinan Jinan 250022 China; ^3^ Laboratory of Molecular Pathology and Cancer Genomics Department of Molecular Medicine and Biopharmaceutical Sciences Graduate School of Convergence Science and Technology Seoul National University Seoul 08826 South Korea; ^4^ RF Bio Center Department of Electronics Engineering Kwangwoon University Seoul 01897 South Korea

**Keywords:** electronic skin, flexible pressure sensor, tactile perception, tactile sensing, wearable sensing

## Abstract

Traditional electronic skins (e‐skins) face significant challenges in system integration and practical usability demonstrations, limiting their ability to meet the development demands of robot intellectualization under future artificial intelligence (AI) frameworks. Here, a bionic dual receptor (BDR) e‐skin motivated by AI‐based hardware‐software coordination is proposed, which consists of electrospinning fiber triboelectric unit inspired by fast‐adapting receptors and micropyramid ionic hydrogel iontronic unit inspired by slow‐adapting receptors. Benefiting from the iontronic effect and micropyramid structure, the iontronic unit yields impressive features: a linear sensitivity of 172 kPa^−1^ (30 kPa), a fast response/recovery time of 11.2 ms. Based on this innovation, the BDR e‐skin is integrated into the glove, and a dual‐channel signal‐motivated intelligent glove cognitive system for sign language gesture identification and robot interaction is developed, laying the foundation for subsequent usability demonstrations. Further, the BDR e‐skin is deeply integrated with intelligent software algorithms and high‐speed hardware circuits to construct an intelligent autonomous material cognition system. This system enables the robot finger controlled by the intelligent glove to accurately identify multidimensional properties (average accuracy: 99.3%) of smooth surface films—such as electronegativity, softness/hardness, and material species—with a single touch, showing a tactile cognition level comparable to that of humans.

## Graphical abstract

In this work, bionic dual receptor (BDR) e‐skin based on the iontronic and triboelectric effects is proposed, inspired by the slow‐adapting and fast‐adapting receptors. Motivated by intelligent software algorithms and high‐speed hardware circuits, the proposed intelligent autonomous material cognition system integrated with the EDB e‐skin can accurately perceive the electronegativity, softness/hardness, and material species within a single touch.

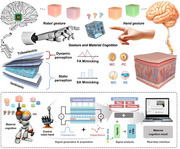

## 1 Introduction

Human skin contains two primary types of mechanoreceptors, namely slow‐adapting (SA) and fast‐adapting (FA) receptors, which are responsible for perceiving different types of pressure stimuli and play an important role in communication and interaction with the external environment.^[^ ^1^, ^2^, ^3^, ^4^ ^]^ SA receptors, such as Merkel cells, are sensitive to sustained pressure and are capable of detecting stable pressure, continuously transmitting signals under sustained stimuli; FA receptors, including Meissner corpuscles and Pacinian corpuscles, are highly responsive to rapidly changing mechanical stimuli, enabling the perception of light touch and vibration, and are particularly suitable for detecting instantaneous changes in pressure.^[^ ^5^, ^6^, ^7^ ^]^ This sophisticated dual‐receptor system constitutes the efficient pressure‐sensing mechanism of human skin, endowing humans with the ability to acquire tactile perception information. Over the past decade, a series of electronic skins (e‐skins) with bionic SA or FA perception mechanisms have been reported, among which piezocapacitive (conventional capacitive sensing and iontronic sensing) and piezoresistive sensors typically mimic SA receptors,^[^ ^8^, ^9^, ^10^, ^11^, ^12^ ^]^ while triboelectric and piezoelectric sensors primarily mimic FA receptors (see Note S1, Supporting Information, for a more detailed description).^[^ ^13^, ^14^, ^15^, ^16^, ^17^ ^]^ Despite the significant advancements in e‐skins inspired by individual receptor functionalities, there are few studies on e‐skins that take into account the functional characteristics of both receptors.

Tactile cognition is a deeper level of tactile perception, which forms an understanding and cognition of object properties by transmitting tactile perception information to the brain and processing it in the cerebral cortex.^[^ ^18^, ^19^ ^]^ This process relies on the synergy of multiple neural mechanisms and involves the joint work of various brain regions, enabling individuals to effectively perceive and cognize objects in the surrounding environment through touch, and interact and operate with them.^[^ ^20^, ^21^ ^]^ With the rapid evolution of flexible electronics and artificial intelligence technologies, tactile perception and cognition have become indispensable abilities for the intellectualization process of robots.^[^ ^22^, ^23^, ^24^, ^25^ ^]^ Zheng et al. proposed a fully fibrous, permeable, adhesive, and intrinsically stretchable e‐skin and developed a 5‐channel human motion sensing system based on it, which can realize real‐time sign language recognition with the assistance of signal processing and machine learning algorithms.^[^ ^26^, ^27^ ^]^ However, gesture recognition still faces challenges in terms of high real‐time performance, high accuracy and system stability, and needs to be further optimized. Further, to expand the capabilities of electronic skin in the field of multimodal cognition, Li et al.^[^ ^28^ ^]^ reported a hybrid e‐skin consisting of a triboelectric nanogenerator (TENG) and a piezoresistive sensor in series, combining a high‐speed data collector and machine learning to build a material perception system that can identify 12 different materials in real‐time through a single touch. Zhang et al.^[^ ^29^ ^]^ proposed a 3D architected e‐skin, whose force and strain sensing components are arranged in a 3D layout, and developed a tactile system that can sense the modulus/curvature changes of objects through touch, thereby identifying the freshness of food. Although these studies have made notable progress in identifying the basic properties of objects, existing technologies still lack a deep understanding and integration of multidimensional tactile information, making it difficult to achieve more autonomous cognition and decision‐making, and still rely on human assistance. Therefore, enhancing the autonomous learning abilities and usability demonstrations of these systems in intelligent perception and cognition is urgent, which is also the key challenge in advancing robot intellectualization.

**Figure 1:**
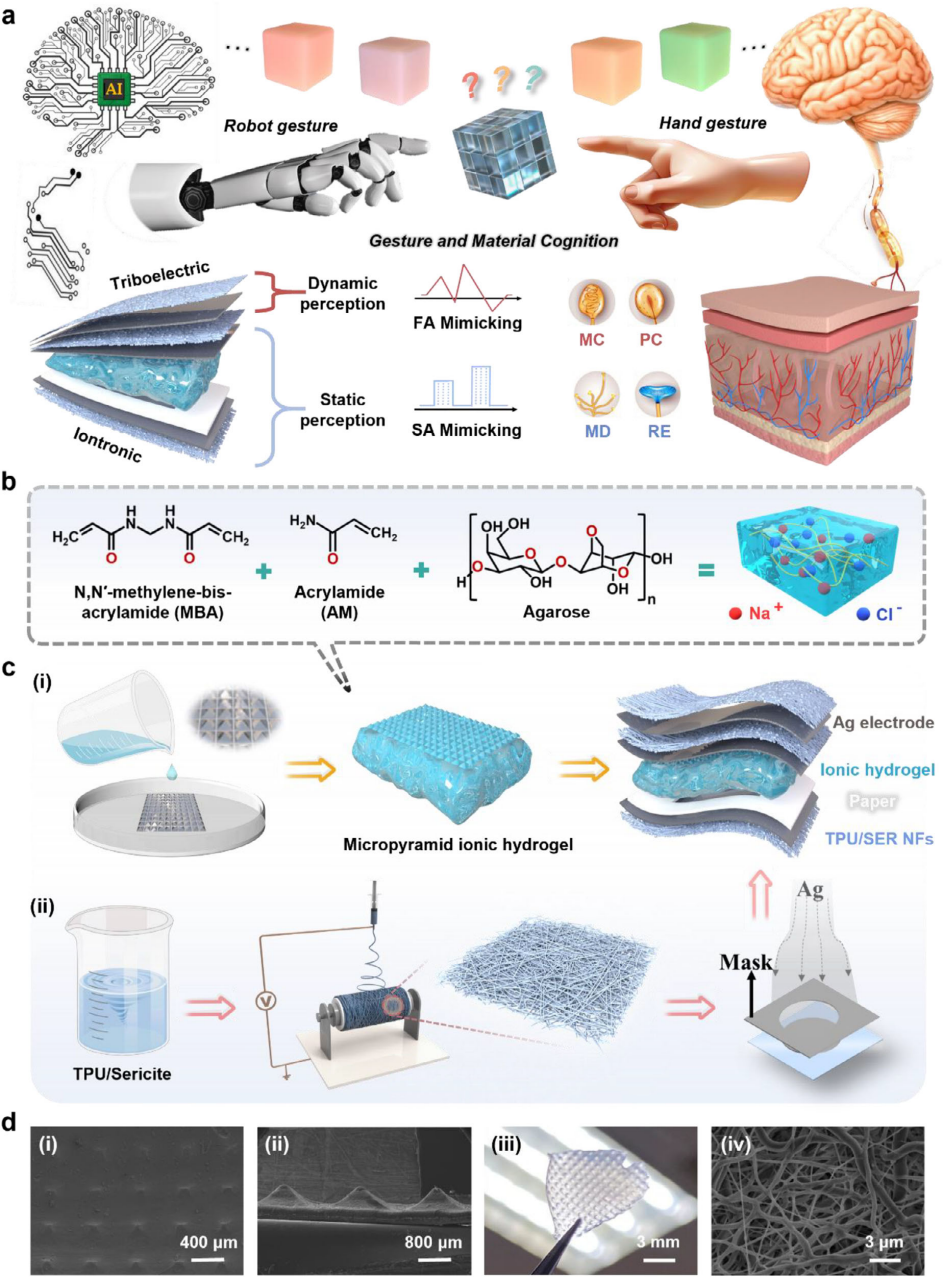
a) Construction drawing of the intelligent tactile cognition system based on BDR e‐skin inspired by the SA‐FA Receptor. b) Schematic of the chemical structures of the three key components of the ionic hydrogel. c) Preparation process and the overall structure of the BDR e‐skin. d) SEM images of micropyramid ionic hydrogel (i, ii), photo of micropyramid ionic hydrogel (iii), SEM image of TPU/sericite fiber membrane (iv).

Here, we propose a bioinspired SA‐FA bionic dual‐receptor (BDR) e‐skin that achieves enhanced multidimensional perception abilities through the synergistic enhancement of static and dynamic stimuli. Further, it is equipped with an “artificial brain” to enable higher‐level intelligent tactile cognition (**Figure** **1**a). The BDR e‐skin consists of two stacked components: an iontronic unit based on micropyramid ionic hydrogel to mimic SA receptors; a triboelectric unit composed of electrospinning thermoplastic polyurethane (TPU)/sericite fibers to mimic FA receptors. The proposed iontronic unit exhibits a linear sensitivity of up to 172 kPa^−1^ (30 kPa), a fast response/recovery time of 11.2 ms, and a detection limit as low as 0.5 Pa. To validate the potential of BDR e‐skin for autonomous cognition and usability demonstrations, the following two specific applications are explored: i) The synergistic enhancement of static perception (iontronic effect) and dynamic perception (triboelectric effect), combined with a deep neural network (DNN), to develop an intelligent glove cognition system motivated by capacitance and voltage dual channels that achieves barrier‐free communication between signers and nonsigners (average accuracy: 99.3%) and supports interactive control of robot hands. ii) By integrating BDR e‐skin with the robot control system, 1D‐convolutional neural network (1D‐CNN), and various functional modules, an intelligent system with autonomous cognition is constructed. This system requires only a single touch by the robotic fingers, controlled via the intelligent glove, to accurately identify the electronegativity, softness/hardness, and material species of six smooth surface films. The average accuracy is 96.2%, and the results are displayed in real‐time on a visual interface.

## 2 Results and Discussion

### 2.1 Structural Design and Characterization

Figure 1b illustrates a schematic of the one‐pot synthesis process for the acrylamide (AM)/agarose/NaCl ionic hydrogel. In this system, NaCl not only provides free Na^+^ and Cl^−^ ions for the hydrogel but also significantly improves the material properties through multifunctional mechanisms. On the one hand, Na^+^, as a mobile ion, significantly enhances the electrical performance of the hydrogel. On the other hand, Na^+^ forms metal‐ion coordination with functional groups within the gel network, thereby greatly enhancing the structural stability and mechanical strength of the hydrogel.^[^ ^30^ ^]^ In addition, AM, as the main monomer, forms a 3D polymer network through cross‐linking polymerization, providing basic network structure and mechanical support for the hydrogel, while its hydrophilic groups facilitate the migration of ions; agarose, as a natural polymer, uses its own chain structure to interweave with the AM network, which not only improves the mechanical strength and structural stability of the hydrogel, but also forms additional physical cross‐linking points during the cooling and gelling process of the solution, giving the material good self‐support and elasticity. The preparation process of the BDR e‐skin is shown in Figure 1c (see the “Experimental Section” for preparation details); its design can be regarded as a vertically stacked architecture comprising an iontronic unit and a triboelectric unit. In the dielectric layer design of the iontronic unit, the significant enhancement of sensing performance by microstructures is fully considered. A commercial tablet packaging board with an anti‐micropyramid structure is selected as the template (Figure S1, Supporting Information), offering advantages such as low cost and scalability for mass production, and successfully achieving high‐efficiency pattern transfer through the ionic hydrogel substrate (Figure 1c‐i). Figure 1d‐i,ii shows the scanning electron microscopy (SEM) images of micropyramid ionic hydrogel, including top‐view and cross‐sectional views. It can be observed that micropyramid structures are uniform and well organized, providing a crucial foundation for achieving high‐performance and stable sensing functions (see Note S2, Supporting Information, for a more detailed description of the size comparison of pyramid structures). The photo of micropyramid ionic hydrogel is shown in Figure 1d‐iii, highlighting its highly transparent characteristics. For the triboelectric unit design, sericite‐doped TPU is selected to significantly enhance the relative dielectric constant and electropositivity of the triboelectric layer. To further increase surface charge density, electrospinning is employed to process the TPU/sericite composite material, successfully preparing fiber membranes with an ultra‐large surface area. The corresponding SEM characterization, as shown in Figure 1d‐iv, illustrates the uniformity and high surface area of the fiber membrane. Finally, elemental analysis of the fiber membrane is conducted by the energy dispersive spectrometer (EDS), as depicted in Figure S2 (Supporting Information). The results reveal the rich C, N, and O elements, confirming TPU as the primary fiber component, while the presence of Al and Si elements indicates the successful preparation of the TPU/sericite fiber membrane. In addition, the basic physical properties and material selection basis of AM/agarose/NaCl ionic hydrogel, Ag electrode, and TPU/sericite fiber membrane are summarized, as shown in Table S1 and Note S3 (Supporting Information).

### 2.2 Piezocapacitive Characteristics of the Device

**Figure 2:**
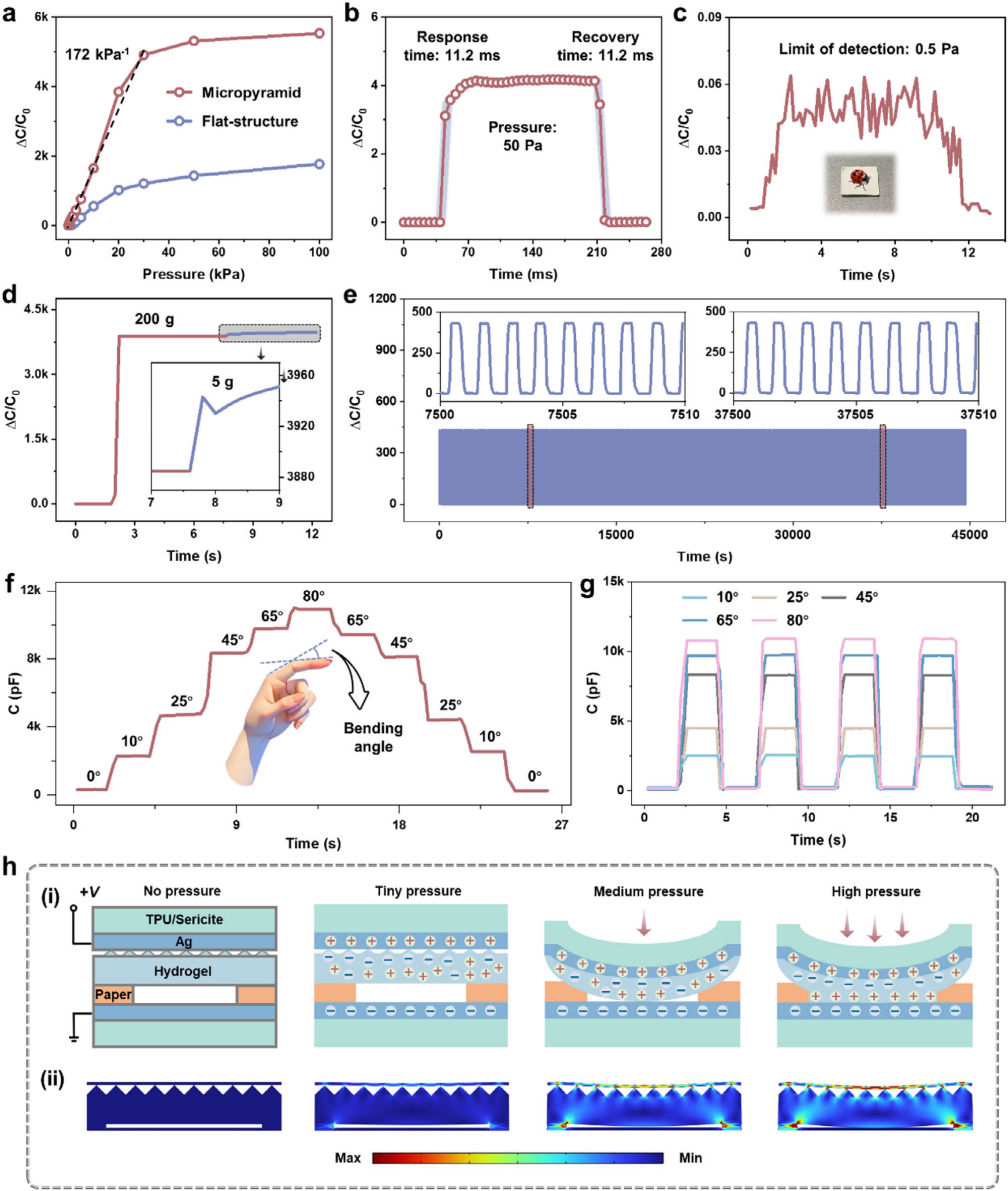
Piezocapacitive characteristics of the BDR e‐skin. a) Capacitance response in iontronic units composed of micropyramid ionic hydrogel and flat‐structure ionic hydrogel under different pressures. b) Capacitance response and recovery curve of the iontronic unit. c) Limit of detection. d) Detection of small load changes under the high load. e) Fatigue testing of the iontronic unit under 38000 pressure cycles. f,g) Sequential and repetitive testing at different bending angles. h) Schematic of the structural changes under different pressure and stress distribution results of the iontronic unit with micropyramid under different pressures.

Since the BDR e‐skin is composed of iontronic and triboelectric units, we conduct a detailed analysis of their respective pressure‐sensing characteristics. First, the pressure‐sensing characteristics of the iontronic unit are investigated and discussed. To this end, the flat‐structure iontronic unit is prepared and compared with the micropyramid iontronic unit, and the capacitance response of the two structures under different pressures is recorded. As shown in **Figure** **2**a, the sensitivity of the micropyramid iontronic unit is significantly higher than that of the flat‐structure, reaching up to 172 kPa^−1^ within a pressure range of 30 kPa, and a wide pressure detection range of 100 kPa is observed. This remarkable enhancement can be attributed to the geometric advantages of the micropyramid structure, which effectively concentrates stress. In addition, the effects of paper spacer layers of different thicknesses on the sensing performance are compared, as shown in Figure S3a (Supporting Information). It can be seen that compared with the devices with 100 and 300 µm spacer layers, the 200 µm spacer layer achieves a good balance between sensitivity, linear range, and detection limit, showing the best performance (see Note S4, Supporting Information, for a more detailed explanation of the comparison of spacer layers of different thicknesses). Figure S3b (Supporting Information) evaluates the repeatability under different pressures. It can be observed that with the increase of external pressure, the capacitance response of the micropyramid iontronic unit exhibits a significant upward trend, with stable and repeatable waveforms during multiple loading‐unloading cycles. Subsequently, the response/recovery time of the micropyramid iontronic unit is tested, which is also a key parameter for measuring pressure‐sensing characteristics. As shown in Figure 2b, the capacitance response of the device rapidly increases to a peak value within 11.2 ms after the pressure is applied, and recovers to the initial level in 11.2 ms after the pressure is removed, which is much faster than recent reports (Table S2, Supporting Information). The fast response/recovery behavior is mainly attributed to the introduction of microstructures, especially the micropyramid structure design. These microstructures can accelerate the storage and release of energy during elastic deformation and recovery. When pressure is applied, the micropyramid structure is compressed, resulting in a fast change in the contact area between the electrode and the ionic gel, which in turn promotes ion migration and accelerates the formation of the electric double layer (EDL), thereby achieving a fast change in capacitance response. After the pressure is released, the micropyramid can quickly return to its original state, ensuring the fast recovery of the iontronic unit. Meanwhile, the limit of detection of the micropyramid iontronic unit is as low as 0.5 Pa (Figure 2c), far superior to that of human skin (≈100 Pa), demonstrating its potential application value in low‐pressure sensing. Based on the comprehensive sensing performance mentioned above, a detailed comparison with high‐level work in recent years is shown in Table S2 (Supporting Information). It can be seen that the micropyramid iontronic unit has several competitive advantages in comprehensive sensing performance. In addition, the ability to detect small pressure changes at high‐pressure levels is also critical. As shown in Figure 2d, when an additional weight of 5 g is applied on top of a preloaded 200 g weight, the capacitance response of the device can still be distinguished, indicating that it has an excellent ability to distinguish small pressures. Figure 2e shows the stability of the device after 38 000 pressure loading‐unloading cycles (12.5 h). It can be observed that the capacitance response does not show significant attenuation during the cycle, proving that it has excellent repeatability, stability, and mechanical durability. Further, the performance of the micropyramid iontronic unit under mechanical bending conditions is investigated. By attaching the device to a flexible plastic substrate, capacitance responses at different angles (10°, 25°, 45°, 65°, and 80°) are monitored in turn (Figure 2f), and the bending repeatability test is performed for each angle (Figure 2g). The results reveal that the device exhibits a stable capacitance response under different bending conditions, further verifying its application potential in bending and deformation scenarios.

The above‐mentioned excellent pressure‐sensing characteristics are mainly attributed to the synergistic effect of the EDL effect and the micropyramid structure. Figure 2h‐i shows the geometric model of the iontronic unit, in which numerous free‐moving Na^+^ and Cl^−^ are distributed within the micropyramid ionic hydrogel. When external pressure is applied, the micropyramid ionic hydrogel contacts the fiber Ag electrode, causing the electrons on the electrode surface to attract ions with opposite charges in the ionic hydrogel. These ions accumulate at the interface between the micropyramid ionic hydrogel and the fiber Ag electrode to form a highly localized EDL.^[^ ^31^, ^32^, ^33^ ^]^ To further elucidate the influence mechanism of the micropyramid structure on the sensitivity, we perform finite element analysis (FEA) based on the COMSOL Multiphysics platform to investigate the deformation characteristics of the micropyramid structure under different pressures (see Note S5, Supporting Information, for detailed parameter settings). As shown in Figure 2h‐ii, when no pressure is applied, there are only a few contact points between the electrode and the dielectric interface, resulting in a low initial capacitance, thus ensuring high sensitivity (derived from the sensitivity formula: *S* = δ(Δ*C*/*C*_0_)/δ*P*); as the pressure gradually increases, the micropyramid structure generates a highly concentrated stress distribution at the contact point, making the contact area more likely to change, thereby giving the iontronic unit a higher sensitivity.

### 2.3 Triboelectric Characteristics of the Device

**Figure 3:**
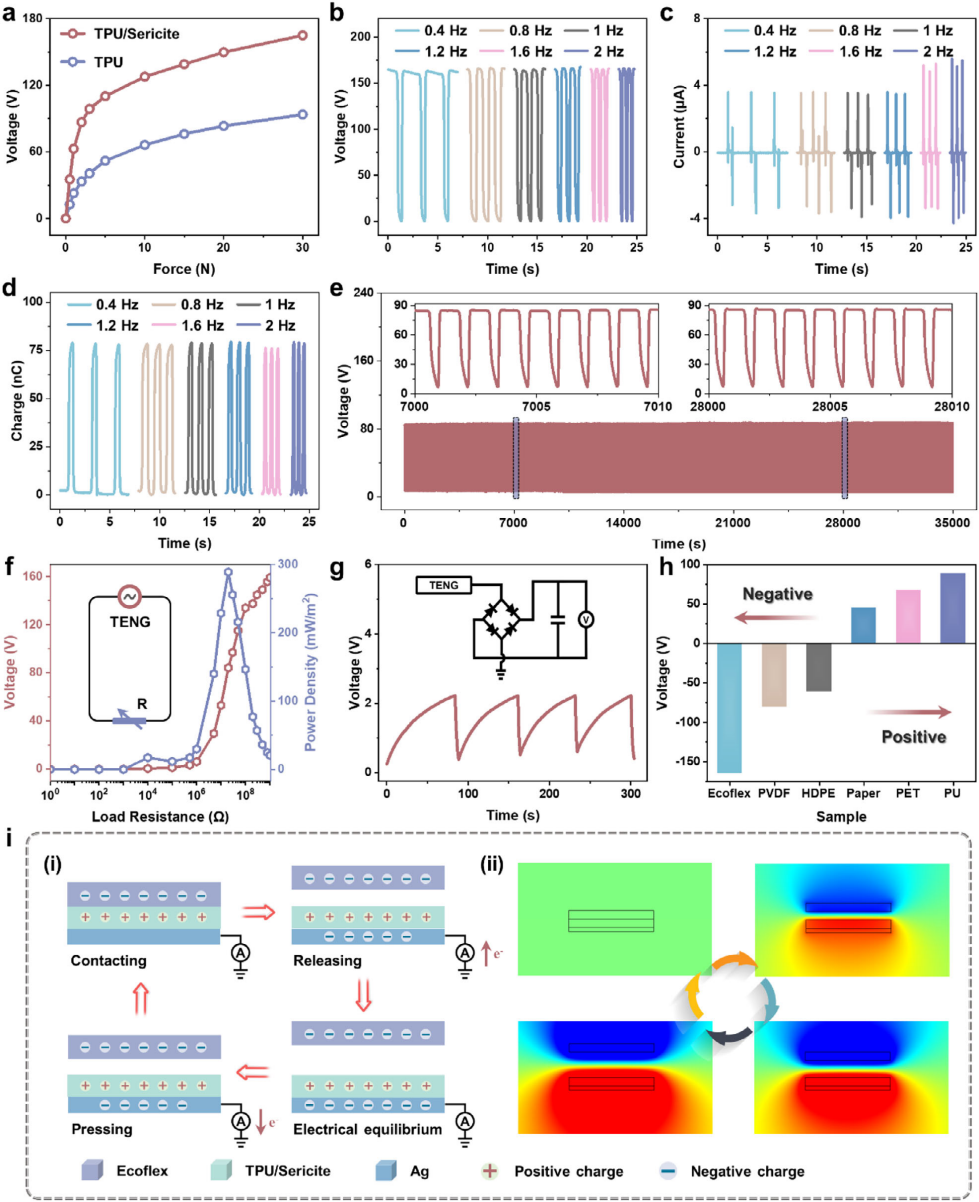
Triboelectric characteristics of the BDR e‐skin. a) Comparison of sensitivity between TPU fiber and TPU/sericite fiber as triboelectric layer. b–d) Response output (open‐circuit voltage, short‐circuit current, and transfer charge) of the triboelectric unit to pressure at different frequencies. e) Voltage output stability test with over 28 000 cycles. f) Changes in output voltage and power density of the triboelectric unit under different external load resistances. g) Charging and discharging curves for a 10 µF capacitor via the triboelectric unit. h) Open‐circuit voltage of the triboelectric unit toward different materials. i) Schematic of the sensing mechanism of the triboelectric unit, and its potential distribution based on COMSOL FEA under different contact‐separation states.

The pressure‐sensing characteristics of the triboelectric unit are introduced as follows, with TPU/sericite serving as the triboelectric layer. **Figure** **3**a shows the sensitivity of triboelectric units with and without sericite‐doped TPU fibers in the range of 0–30 N. Since the doping of sericite significantly enhances the electropositivity of TPU fibers, resulting in higher sensitivity for the TPU/sericite triboelectric unit compared to pure TPU. As shown in Figure 3b–d, under a constant force of 10 N, the open‐circuit voltage, short‐circuit current, and transfer charge of the triboelectric unit are tested at varying frequencies of 0.4, 0.8, 1, 1.2, 1.6, and 2 Hz. It can be observed that with increasing frequency, the open‐circuit voltage and transfer charge exhibit a relatively stable trend, while the short‐circuit current increases progressively, effectively mitigating potential frequency interference in subsequent applications involving intelligent material cognition. Moreover, the output voltage of the triboelectric unit remains stable over more than 28 000 cycles (9.7 h) of alternating loading and unloading pressure, demonstrating excellent durability and repeatability of the proposed triboelectric unit, as depicted in Figure 3e. Further, as a TENG, the proposed triboelectric unit also possesses a self‐powered ability. Figure 3f shows the trend of current and power density under load resistance change, indicating that the current density decreases with the increase of load resistance, and the maximum power density of 288.8 mW m^−2^ is obtained when the load resistance is 20 MΩ. Through the bridge rectifier circuit, the pulse voltage output by the triboelectric unit can be used to charge a commercial 10 µF capacitor. As shown in Figure 3g, the capacitor is quickly charged to more than 2 V in a short time. In addition to the excellent pressure‐sensing characteristics, the triboelectric unit also shows output voltage sensitivity to different contact materials, which stems from the inherent difference in electron affinity between TPU/sericite and the various contact materials.^[^ ^34^, ^35^, ^36^, ^37^ ^]^ As shown in Figure 3h, taking Ecoflex, polyvinylidene difluoride (PVDF), high‐density polyethylene (HDPE), Paper, polyethylene terephthalate (PET), and PU as examples, the voltage output and polarity of the triboelectric unit vary significantly with the different contact materials. Specifically, when TPU/sericite comes into contact with Ecoflex, PVDF, or HDPE, a positive voltage is generated, whereas contact with Paper, PET, or PU results in a negative voltage. In addition, the voltage peaks corresponding to each material are also significantly different. Therefore, by detecting the peak value and polarity of the voltage output of the triboelectric unit, it is possible to effectively infer the species of contact material.

Figure 3i‐i illustrates the sensing mechanism of the triboelectric unit that converts mechanical energy into electrical energy, where Ecoflex serves as the negative triboelectric layer and the TPU/sericite fiber membrane acts as the positive triboelectric layer. When Ecoflex comes into contact with the TPU/sericite fiber membrane, due to their differing electron affinities, equal but oppositely charged electrical charges are generated on each material. In detail, Ecoflex tends to gain charge, while TPU/sericite fiber membrane tends to lose charge, resulting in overall electrical neutrality. When the two materials begin to separate, the positive charges on the TPU/sericite fiber membrane induce negative charges on the Ag electrode due to electrostatic induction. Consequently, negative charges flow from the ground to the Ag electrode, generating a current pulse. As the two triboelectric layers are far enough apart, the charges reach a state of equilibrium again. Finally, with the Ecoflex approaching again until it contacts the TPU/sericite fiber membrane, the negative charge on the Ag electrode flows back to the ground, generating a reverse current pulse, thus forming a complete alternating signal generation cycle. By repeating this contact‐separation cycle, alternating current can be continuously generated. As shown in Figure 3i‐ii, the potential distribution is further analyzed through simulation using COMSOL FEA, which verifies the theoretical model of the contact‐separation mechanism of the triboelectric unit, providing a better understanding of the current generation mechanism. In particular, due to the excellent surface physical properties and porous fiber structure of the TPU/sericite triboelectric layer, reliable contact is achieved between the Ecoflex and the TPU/sericite triboelectric layer upon contact, increasing the relative contact area, effectively promoting the stability and voltage output of the triboelectric unit.

### 2.4 Intelligent Glove Cognition System

**Figure 4:**
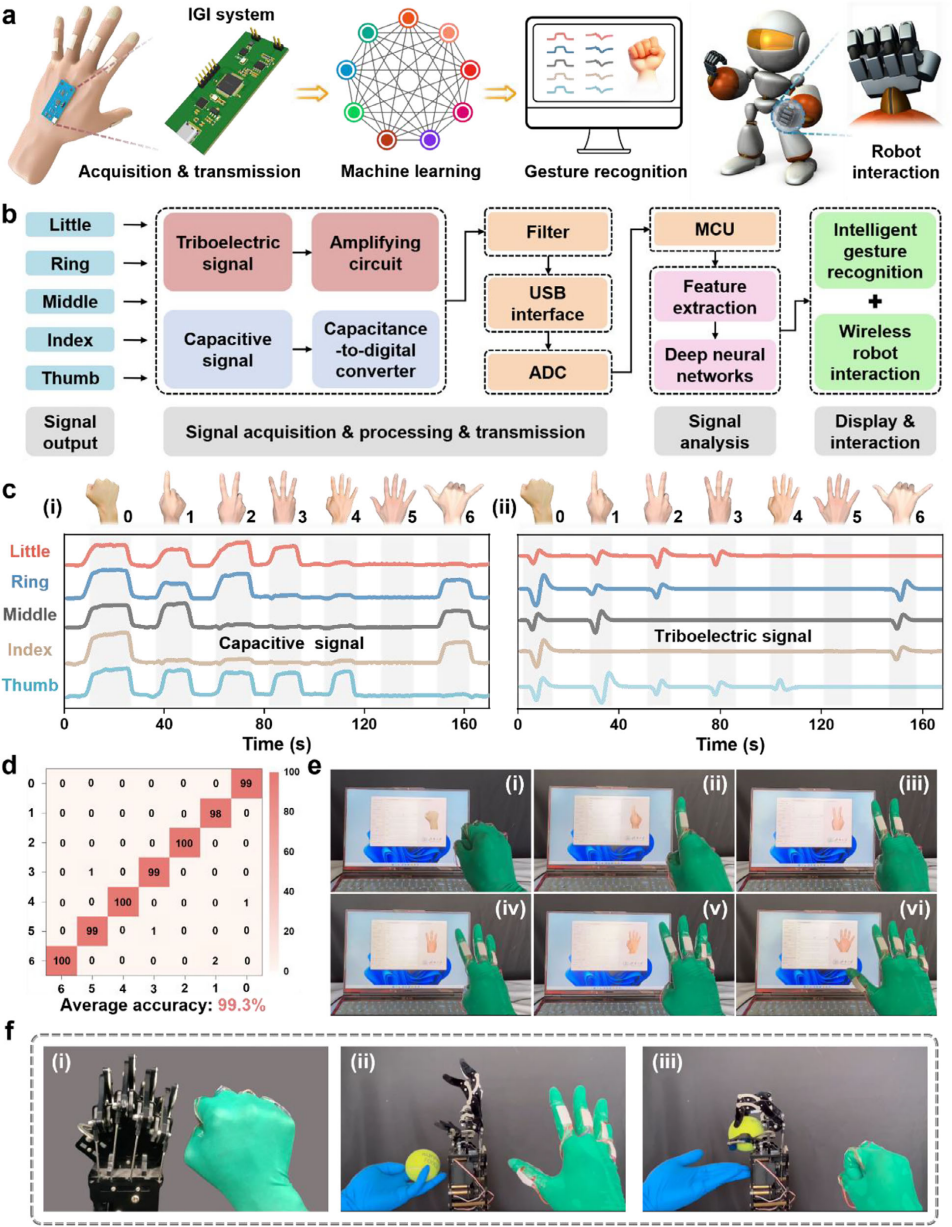
Demonstration of intelligent glove cognition system. a) Concept diagram of the designed intelligent glove cognition. b) Flowchart of the core technical architecture of the intelligent glove cognition system. c) Extracted capacitance and voltage signal waveforms of different sign language gestures. d) Confusion matrix showing classification accuracy (%) for sign language gesture cognition based on capacitance and voltage signals. e) Real‐time display interface for different digital gesture cognition. f) Real‐time interaction between intelligent glove and robot hand.

With the increasing societal demand for barrier‐free communication, sign language, as a vital tool for hearing‐impaired people to communicate with the outside world, has garnered significant attention. However, the popularization and understanding of sign language still face numerous challenges. To this end, we propose an intelligent glove cognition system that integrates sensing technology and machine learning technology. This system can accurately perceive hand movements and gestures, converting them into identifiable information, thereby facilitating barrier‐free communication between signers and nonsigners, and expanding its application in robot interactive control (**Figure** **4**a). The core technical architecture of the intelligent glove cognition system is shown in Figure 4b, including signal output, signal acquisition/processing/transmission, signal analysis, and real‐time display/interaction modules. The system employs BDR e‐skin with excellent flexibility and bending properties, ensuring effective adaptation to finger extension and various bending angles. When the user wears the glove and performs gestures, the triboelectric unit generates voltage signals instantaneously (output via an amplification circuit), while the iontronic unit produces static strain capacitance signals (output via a capacitance‐to‐digital conversion circuit, see Note S6, Supporting Information, for details), which is then filtered through the microcontroller unit (MCU) and transmitted to the host computer (Figure S4, Supporting Information) (see the “Experimental Section” for detailed process). Within the host computer, an embedded DNN extracts and classifies features from the received signals, enabling precise identification of sign language gestures and completing corresponding display and interaction (see the “Experimental Section” for detailed process). Figure 4a,b shows the capacitance and voltage signal waveforms extracted for seven different sign language gestures, each exhibiting unique signal characteristics. To verify the classification performance of the system, the above signal data are input into the DNN for training and testing, and the confusion matrix of the seven sign language gestures based on capacitance and voltage signals are generated, as shown in Figure S5a,b (Supporting Information), with average accuracies of 96.8% and 96.4%, respectively. Further, to improve the accuracy of the system, the capacitance and voltage signals are combined to form a dual‐modal data for model training and learning. The results demonstrate that the combination of dual‐modal signals significantly improves the prediction performance, with the average accuracy reaching 99.3% (Figure 4d) (see Note S7, Supporting Information, for the advantages of dual‐mode signals). Additionally, the intelligent glove system also features real‐time display functionality, as shown in Figure 4e and Figure S6 (Supporting Information). As the sign language gesture gradually changes from the number “0”–“1–6”, the corresponding signal waveforms and matched gesture images are synchronously displayed. Moreover, a dynamic demonstration of real‐time sign language gesture cognition is presented in Movie S1 (Supporting Information). In addition to sign language cognition, the system is also extended to interactive control of robot hands. Through multithreshold identification processing, the collected capacitance and voltage signals are mapped to the driving channels of the robot fingers, enabling precise motion control. Demonstrations have shown that the robot hand can achieve real‐time switching between different sign language gestures and complete complex tasks, including tennis ball grasping, as illustrated in Figure 4f, Figure S7, and Movie S2 (Supporting Information). Undoubtedly, the successful demonstration of this intelligent glove cognition system further confirms the application potential of BDR e‐skin, offering technical possibilities for future barrier‐free communication and robot interactive control.

### 2.5 Intelligent Autonomous Material Cognition System

**Figure 5:**
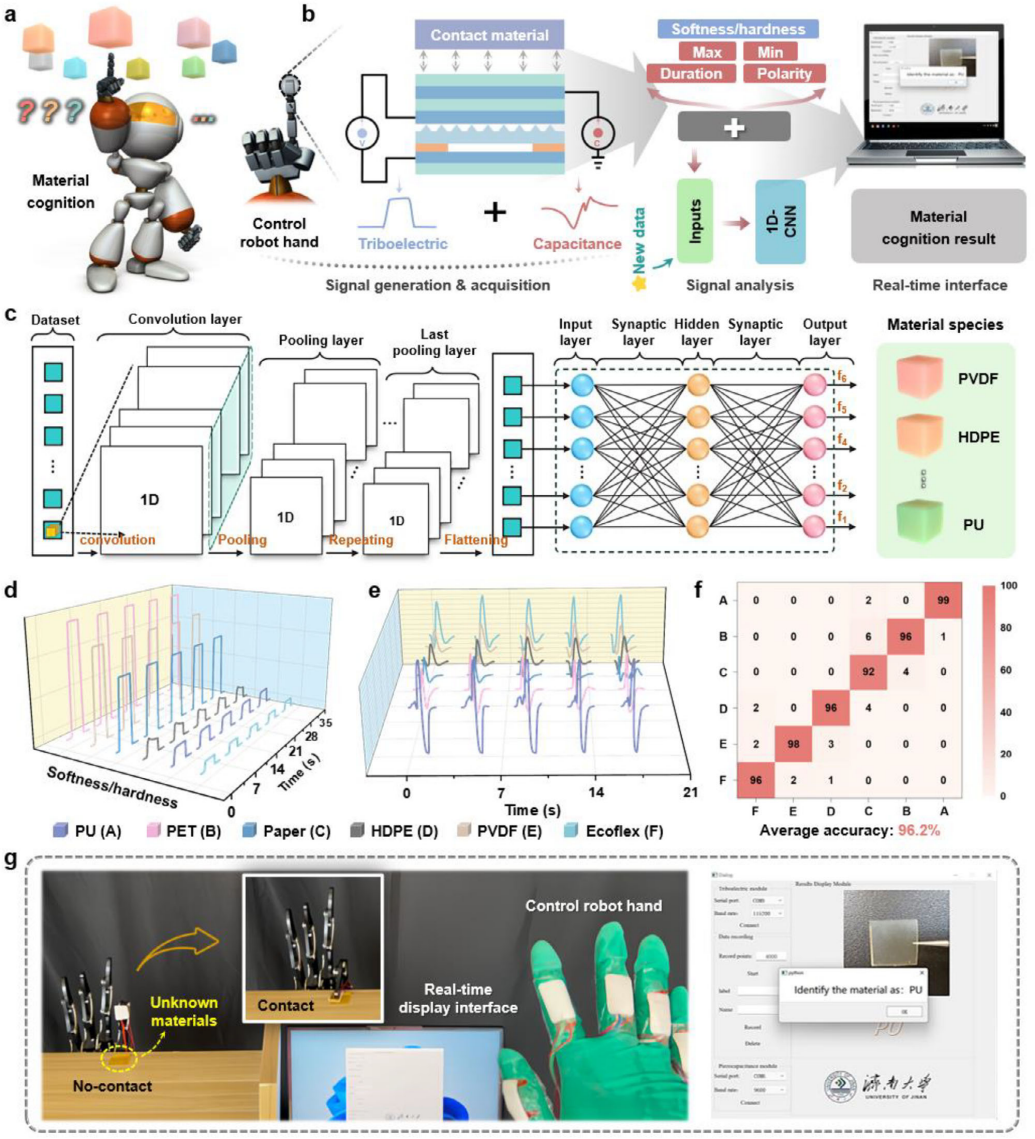
Demonstration of intelligent autonomous material cognition system. a) Concept diagram of the designed intelligent autonomous material cognition. b) Autonomous material cognition process flow, including data generation/acquisition process, signal analysis, and real‐time interface. c) System block diagram of 1D‐CNN. d) Extracted capacitance signal waveforms when in contact with different materials. e) Extracted voltage signal waveforms when in contact with different materials. f) Confusion matrix showing classification accuracy (%) for autonomous material cognition based on capacitance and voltage signals. g) Overview of the application scenario of the constructed intelligent autonomous material cognition system.

Tactile, as a critical sense organ for human perception of the external environment, relies on mechanoreceptors within the skin to convert tactile stimuli into bioelectrical signals, which are then transmitted through neural networks to relevant brain regions for processing and decoding. The process of tactile cognition depends on the deep integration of real‐time neural activity and individual experiential memory, enabling the identification, classification, and cognition of objects. However, for some objects whose surface properties are difficult to distinguish, the cognition of their subtle characteristics remains challenging. To address this, we install BDR e‐skin onto the robot's index finger and develop an intelligent autonomous material cognition system by integrating the robot control system, 1D‐CNN (see Note S8, Supporting Information, for a description of the advantages), and various functional modules (including signal acquisition, transmission, processing, and real‐time display), as shown in **Figure** **5**a. Specifically, 6 materials, including Ecoflex, PVDF, HDPE, Paper, PET, and PU, are selected as the test objects (Figure S8, Supporting Information). When the user controls the bending of the robot's finger through the intelligent glove, the BDR e‐skin on the index finger contacts different materials. The MCU collects dual‐modal signals (capacitance and voltage) generated by the e‐skin and inputs them into the pre‐trained 1D‐CNN model (see Table S3, Supporting Information, for detailed parameters), ultimately obtaining predictions that are displayed in real‐time (Figure 5b,c) (see the “Experimental Section” for detailed process). Under constant pressure conditions, the difference in elastic modulus (softness/hardness) of different materials leads to significantly different capacitance signals when they come into contact with the iontronic unit, as shown in Figure 5d; meanwhile, differences in electronegativity between different materials and the triboelectric layer lead to unique voltage signal waveforms (Figure 5e). Under constant pressure conditions, the difference in elastic modulus (softness/hardness)^[^ ^38^, ^39^, ^40^ ^]^ of different materials leads to significantly different capacitance signals when they come into contact with the iontronic unit, as shown in Figure 5d. In addition, Table S4 (Supporting Information) provides the range values of elastic modulus for six materials for reference. Meanwhile, differences in electronegativity between different materials and the triboelectric layer lead to unique voltage signal waveforms, as shown in Figure 5e and Figure S9 (Supporting Information). Furthermore, the system uses the capacitance and voltage signals of the above six different materials as datasets (a total of 500 groups), of which 80% of the data are used as training sets and 20% as test sets. The samples cover multiple time points and environmental variables to reduce the impact of environmental factors on identification accuracy. Figure S10a,b (Supporting Information) shows the confusion matrix of the classification results of the six materials for static capacitance perception and dynamic triboelectric perception, with average accuracies of 93.7% and 90.7%, respectively. Further, the above two types of signal data are combined into a single dataset, and the final cognition result is shown in Figure 5f, with an average accuracy of up to 96.2%. In addition, due to the signal conditioning at the hardware level, the multidimensional feature modeling at the algorithm level, and the disturbance enhancement at the training level in the system, it still has high material cognition accuracy under different pressures (see Note S9, Supporting Information, for a more detailed explanation). Figure 5g shows the actual demonstration of the intelligent autonomous material cognition system. Taking PU as an example, the robot's index finger can complete autonomous cognition after touching the material surface once, and the results are displayed in real‐time on the computer interface. For additional details on the material cognition process, refer to Figure S11 and Movie S3 (Supporting Information). Additionally, a detailed comparison is made with the literature related to material identification reported in recent years in terms of identification dimensions and system capabilities, as shown in Table S5 (Supporting Information). In the long term, the proposed intelligent autonomous material cognition system exhibits exceptional performance and broad application prospects, positioning it as a potential driving force in advancing robot intellectualization.

## 3 Conclusion

This work reports a BDR e‐skin that integrates triboelectric and iontronic units to mimic mechanoreceptors of human skin. The active layers of the triboelectric and iontronic units are efficiently and cost‐effectively prepared on a large scale using electrospinning and template transfer methods, enabling the mimics of both dynamic and static tactile perception functions of SA and FA receptors. In terms of pressure sensing, the iontronic unit of BDR e‐skin exhibits high sensitivity, rapid response/recovery speeds, and a low limit of detection, all of which are crucial for intelligent applications. To give full play to the dynamic and static perception abilities of the BDR e‐skin, an intelligent glove cognition system that integrates iontronic (capacitance channel) and triboelectric (voltage channel) effects is first constructed for the demonstration of sign language cognition and robot interactive control. Further, leveraging this robot control system and integrating software algorithms with hardware circuits, an intelligent autonomous material cognition system is developed. This system, controlled by the intelligent glove, allows the robot's index finger to identify material species based on a single touch and display the cognition results in real‐time, demonstrating cognition abilities comparable to those of humans. However, the system's stability in complex environments and its ability to work continuously for a long time remain to be verified; in the future, it will need to be optimized in terms of durability, environmental adaptability, and expansion to more material categories, and explore online self‐learning and multimodal feedback integration. Clearly, this work provides innovative ideas for the application of e‐skin in autonomous cognition and feedback in robots, showcasing its broad potential for use in intelligent prosthetics, the metaverse, and military fields.

## 4 Experimental Section

### Materials

Agarose, AM (≥99.9%), ammonium persulfate (APS, ≥98%), N,N,N’,N’‐tetramethylethylenediamine (TMEDA), N,N’‐methylene‐bis‐acrylamide (MBA, ≥99%), N,N‐dimethylformamide (DMF), tetrahydrofuran (THF, ≥99.5%), glycerol (≥99.5%), and NaCl (≥99.5%) were purchased from Shanghai Aladdin Biochemical Technology Co., Ltd. Sericite and TPU were purchased from Taobao Co., Ltd, Alibaba Group, China. The volunteer participating in the test voluntarily wore cleaned nitrile gloves under laboratory conditions with full informed consent. No direct skin contact occurred during the study, and therefore, this does not constitute human subject research and does not require ethical review.

### Preparation of TPU/Sericite Fiber Membrane

The 0.15 g of sericite powder was transferred into a reagent bottle, and then 6.74 g of DMF solution and 13.48 g of THF solution were added dropwise using a dropper, followed by 30 min of ultrasonic dispersion to make the sericite uniformly dispersed in the solution. Subsequently, 1.85 g of TPU particles were added to the prepared sericite solution, and magnetic stirring was performed at 80 °C with a rotation speed of 800 rpm until the TPU dissolved. The resulting precursor solution was then filled into a syringe for electrospinning, the solution propulsion speed was set to 1 mL h^−1^, the voltage was set to 18 kV, the distance between the collector and the spinneret was kept at 12 cm, and the speed of the collecting wheel was set to 200 rpm. After the electrospinning process was completed, the fiber membrane was removed and dried in an oven at 80 °C for 5 min.

### Preparation of TPU/Sericite/Ag Fiber Membrane Electrode

Ag was deposited onto the TPU/sericite fiber membrane via direct current sputtering using an ultra‐high vacuum magnetron sputtering deposition system (see Table S6, Supporting Information, for detailed experimental parameters).

### Preparation of AM/Agarose/NaCl Ionic Hydrogel

The 1.25 g of AM, 10 mg of MBA, and 50 mg of agarose were added into a reagent bottle. Then, 5 g of deionized water was added, and the mixture was stirred magnetically at 120 °C and 800 rpm for 20 min until the solution became clear. After cooling, 10 mg of APS, 100 mg of NaCl, and 2.5 µL of TMEDA were added to the prepared solution. Using a commercial tablet packaging board with aiti‐micropyramid structure as a template, the solution was dropped onto the template with a dropper. The template was then placed in an 80 °C oven and heated for 5 min to allow the ionic hydrogel to solidify. The ionic hydrogel was carefully peeled off the template to obtain micropyramid structure, which was placed in a cold environment at 5 °C for 30 min to facilitate the formation of a double‐network structure. Finally, the ionic hydrogel was immersed in glycerol for 30 min, where solvent exchange occurred to maintain its water content.

### Assembly of BDR e‐Skin

First, the TPU/sericite/Ag fiber membrane electrode (two layers), the AM/agarose/NaCl ionic hydrogel, the isolated layer of hollow square‐shaped paper, and the Ag/TPU/sericite fiber membrane electrode were stacked from bottom to top and encapsulated with the VHB tape (VHB‐4905, 3M). Then the conductive tape was used to lead out the wires from electrodes, respectively, thus successfully constructing the BDR e‐skin.

### System Operation Flow of Acquisition/Processing/Transmission of Capacitance and Triboelectric Dual‐Mode Signals

The system uses the STM32 series MCU as the core control unit, which has rich analog and digital interface resources and can stably realize multichannel data acquisition, preprocessing and communication with the host computer. The triboelectric signal channel is used to respond to rapid mechanical stimulation. The signal was first conditioned by a set of low‐noise differential amplifiers and RC filter circuits, and then sampled by the ADC inside the MCU. It has high voltage response sensitivity and frequency adaptability, and can effectively extract the pulse signal characteristics of triboelectric output. The capacitance signal channel is responsible for recording continuous static pressure changes. It uses the high‐precision capacitance‐to‐digital converter FDC2214 chip for measurement. It has multichannel input, automatic calibration and high‐resolution (down to fF level) sampling capabilities to ensure high stability and high‐precision detection of tiny capacitance changes in ionic hydrogel media. The two signals correspond to the analog FA and SA tactile mechanisms respectively. After being packaged and integrated by the MCU, they are transmitted to the host computer through the serial port to achieve efficient coupling with the embedded DNN or 1D‐CNN model.

### Algorithm Construction of Intelligent Glove Cognition System

The MCU synchronously collects the triboelectric voltage signal (analog signal amplified by operational amplifier) and the capacitance signal (converted to digital value by CDC), and packages the data in a dual‐channel signal window of ≈300–500 ms for each gesture, and uploads it to the host computer through serial communication (UART protocol). In the host computer, the signal is first processed by a Python script, including Z‐score normalization, sliding window segmentation, and feature compression; then, the processed data is sent to the three‐layer fully connected neural network we built for classification. The network input layer is dual channel × time step (such as 2 × 50), contains two hidden layers (ReLU activation), the number of neurons is 128 and 64, respectively, and the output layer is a Softmax classifier, which can recognize six types of sign language gestures. The model uses Scikit‐learn and TensorFlow platforms, uses the Adam optimizer, the learning rate is 0.001, and the training round is 300 rounds, with a cross‐validation accuracy of 99.3%. The final model is exported in.h5 format and called in the Python host computer interface to implement real‐time reasoning. The average recognition time per frame is ≈10–20 ms, fully meeting the real‐time requirements.

### Algorithm Construction of Intelligent Autonomous Material Cognition System

Taking the dual‐channel time series of capacitance and triboelectric signals as input, 1D‐CNN is used for end‐to‐end feature extraction and classification. The network structure is: input dimension is 2 × 50; Conv1D layer has 64 filters, kernel size 3, step size 1, and activation function is ReLU; followed by a Pooling layer with a pooling window of 2; Flattening enters the fully connected layer, which contains 128 neurons and uses Dropout 0.5 to prevent overfitting; the final output layer uses Softmax to classify six types of materials. The model is based on 500 sets of experimental data (80% for training and 20% for testing), covering different time periods and room temperature environments, and the test accuracy is 96.2%. After training, the model is saved in the host computer, receives sensor data in real time, and completes multidimensional cognition such as material type, hardness, and electronegativity. The prediction results are displayed in the visual interface, and the average response time is ≈30–50 ms.

## Conflict of Interest

The authors declare no conflict of interest.

## Author Contributions

H.L. and H.N. conceived this work. H.L. and Y.L. designed, prepared, and tested the e‐skin. H.L. and H.N. developed the overall system and verified the feasibility of this architecture. H.L., H.N., and Y.L. helped with the experiments design and system preparation. H.K., H.L, E.S.K., and N.Y.K. participated in the discussion of experimental results. H.L., H.N., and Y.L. wrote the manuscript. Y.L. supervised the project. All authors reviewed and commented on the manuscript.

## Supporting information



Supporting Information

Supplemental Movie 1

Supplemental Movie 2

Supplemental Movie 3

## Data Availability

The data that support the findings of this study are available from the corresponding author upon reasonable request.
