# Supplementary material for: Bio‐Inspired SA‐FA Bionic Dual Receptor Electronic Skin for Intelligent Gesture and Material Cognition Systems Enhanced by Static‐Dynamic Mutual Interaction
Source: Adv Sci (Weinh). 2025 Aug 13;12(41):e09740. doi: 10.1002/advs.202509740 (PMC12591205; doi:10.1002/advs.202509740)
Supplement: Supplementary file 1 — Supporting Information [file ADVS-12-e09740-s003.docx]

Supporting Information

**Bio-Inspired SA-FA Bionic Dual Receptor Electronic Skin for Intelligent Gesture and Material Cognition Systems Enhanced by Static-Dynamic Mutual Interaction**

*Hao Li, Hongsen Niu^*^, Hao Kan, Eun-Seong Kim^*^, Nam-Young Kim^*^, Yang Li^*^*

H. Li, Prof. Y. Li

School of Integrated Circuits

Shandong University

Jinan, 250101, China

E-mail: yang.li@sdu.edu.cn

Prof. H. Niu, Prof. H. Kan

Shandong Provincial Key Laboratory of Ubiquitous Intelligent Computing

School of Information Science and Engineering

University of Jinan

Jinan, 250022, China

E-mail: ise_niuhs@ujn.edu.cn

Prof. E.-S. Kim, Prof. N.-Y. Kim

Laboratory of Molecular Pathology and Cancer Genomics

Department of Molecular Medicine and Biopharmaceutical Sciences

Graduate School of Convergence Science and Technology

Seoul National University

Seoul, 08826, South Korea

E-mail: 3037eskim@gmail.com

Prof. E.-S. Kim, Prof. N.-Y. Kim

RF Bio Center

Department of Electronics Engineering

Kwangwoon University

Seoul, 01897, South Korea

E-mail: nykim@kw.ac.kr

Corresponding authors: Prof. Hongsen Niu, Prof. Eun-Seong Kim, Prof. Nam-Young Kim, Prof. Yang Li

**Supplementary Note
Note S1. Classification criteria for simulating different receptor types using sensors.**

In terms of response characteristics analogy: (1) SA receptors are characterized by their high sensitivity and stable output to continuous pressure, which is highly consistent with the response characteristics of piezocapacitive and piezoresistive sensors when a constant load is applied --- the capacitance or resistance will change significantly and linearly, and the signal output can be maintained for a long time. (2) FA receptors mainly respond to rapidly changing stimuli, and their output decays transiently. Similarly, triboelectric and piezoelectric sensors only generate pulse charge or voltage peaks at the moment of contact/separation or loading/unloading. Under continuous pressure, the signal decays rapidly or even returns to zero, showing a typical “fast adaptation” characteristic.

Correspondence of electrical mechanisms: (1) piezocapacitive/piezoresistive sensors rely on the steady-state change of the contact area/channel cross-sectional area between the electrode and the dielectric (or conductive network). Their response is proportional to the applied pressure, and they have the ability to continuously monitor static loads, which is consistent with the “slow adaptation, continuous response” characteristics of SA receptors. (2) Triboelectric/piezoelectric sensors are based on the transient process of charge separation or internal polarization (crystal). They only generate electrical signals when contact-separation or stress changes, and the signal amplitude is related to the rate of change, which is suitable for simulating the “fast adaptation, dynamic change perception” function of FA receptors.

Significance in biological function simulation: (1) Using piezocapacitive/piezoresistive sensors to simulate SA receptors helps capture stable “static tactile” information such as object deformation and pressure distribution; (2) Triboelectric/piezoelectric sensors are more suitable for simulating FA receptor functions and capturing rapidly changing “dynamic tactile” information such as vibration, touch, and pulse; this combination enables the artificial system to better reproduce the dual-mode tactile mechanism of SA receptors and FA receptors in human skin that synergistically perceive static and dynamic stimuli.

**Note S2. Comparison of the advantages of pyramid structures of different sizes.**

Accurate control of microstructure size is crucial to the sensing performance of e-skin. The micropyramid structure significantly increases the surface area through high-precision patterning, thereby improving the response sensitivity of the ionic hydrogel to tiny external stimuli. Under a scanning electron microscope (SEM), it can be observed that the height, top angle, and base side length of the micropyramid are highly uniform and regular, which is crucial for ensuring the performance consistency of the entire device.

When the size of the pyramid unit is further reduced to the nanoscale, its surface area per unit volume increases dramatically, thus showing higher sensitivity to low-voltage signals; however, nanoscale structures are prone to defects or incomplete replication during the preparation process, and their own stability is insufficient, which may cause the surface of the ionic hydrogel to break under repeated loading. Relatively speaking, when the size of the pyramid unit is enlarged to the millimeter range, the structural durability and wear resistance are improved, but the relative reduction in contact area leads to a decrease in sensing sensitivity and increased energy loss, thereby weakening the electronic skin's ability to detect weak pressure changes.[1-3]

Therefore, this study selected the micron-scale pyramid size in the design to achieve the best balance between sensing performance and mechanical stability: this size can provide sufficient effective surface area to capture tiny pressure signals, while ensuring the integrity and repeatability of the structure in repeated deformation. In addition, the micron-scale structure can be prepared on a large scale through mature processes such as template transfer, with low cost and high yield, laying the foundation for the industrial application of e-skin.

**Note S3. Basis for material selection.**

AM/agarose/NaCl ionic hydrogel: The ionic hydrogel selected in this manuscript has excellent ionic conductivity and flexibility, can efficiently conduct weak electrical signals, and is particularly suitable for static capacitive sensing modules. In addition, the hydrogel exhibits good mechanical toughness and biocompatibility at room temperature, providing a strong guarantee for the long-term stable operation of flexible electronic devices.

Ag electrode: To ensure efficient transmission of electrical signals, we use high-purity Ag materials. Ag not only has excellent conductivity but also exhibits good electrical stability and adhesion to the substrate, making it easier to obtain consistent and reliable electrode morphology during the micro-nano structure patterning process.

TPU/sericite fiber membrane: The composite nanofiber membrane prepared by electrospinning has flexibility, elasticity, and porous network structure. On the one hand, it provides a solid mechanical support for the upper triboelectric nanostructure; on the other hand, the porous structure helps to adjust the contact area, thereby improving the repeatability and stability of the triboelectric signal.

**Note S4. Comparative explanation of the sensing performance of spacer layers with different thicknesses.**

The paper spacer is located between the bottom electrode and the ionic hydrogel, and its thickness change will directly affect the sensitivity, linear range, and detection limit of the device. Specifically, as the thickness of the paper increases, excessive thickness will limit the amount of compression after compression, reducing ΔC, thereby affecting the overall linear range and detection limit. When the thickness of the paper decreases, too small a thickness can increase ΔC under slight pressure, thereby improving the high response of the device to weak pressure. But at the same time, due to the thin spacer, the relative sensitivity is more likely to have the “early contact” phenomenon during the compression process, resulting in the early appearance of the nonlinear interval of the response curve and limited detection range.

**Note S5. Detailed parameter settings for the COMSOL FEA simulation.**

In the solid mechanics module, the TPU/Ag fiber membrane electrode and the AM/agarose/NaCl ionic hydrogel dielectric layer are defined as isotropic linear elastic materials, with Young’s modulus E = 4.5 MPa and Poisson’s ratio ν = 0.35 for the former and E = 30 kPa and ν = 0.4 for the latter. The electrode-hydrogel interface adopts penalty function contact (penalty factor fp = 1), a fixed constraint is applied to the lower surface of the bottom electrode, a pressure load is applied to the upper surface of the top electrode, and the remaining boundaries are free. The mesh selects refined units controlled by the physical field. In the electrostatic field module, the electrode and dielectric materials are set as charge conservation materials, the electrolyte model maintains relative node constants, the relative dielectric constant ε = 6.7 and the conductivity σ = 3.99 × 10^5^ S/m of the TPU/Ag fiber membrane, the ionic hydrogel ε = 65 and σ = 5 S/m, the air domain boundary is set to zero charge condition, and each parallel electrode is applied with a constant potential of 0 V (ground) and 1 V, respectively.

**Note S6. Construction of capacitance-to-digital conversion circuit.**

The FDC2214EVM module based on the LC resonant circuit principle amplifies the capacitance signal, where the input end of each detection channel of the module is connected to an inductor and a capacitor to form an LC circuit. The LC circuit end is connected to the iontronic unit, which will generate an oscillation frequency. The value of the measured capacitance can be determined by calculating this frequency value, realizing the conversion of the capacitance analog quantity to the digital quantity, and the final result is sent to the MCU end through the inter-integrated circuit.

**Note S7. Advantages of dual-mode signals.**

Dual-modal signal fusion can give full play to the response advantages of the static capacitance channel and the dynamic triboelectric voltage channel to different features, thereby significantly improving the overall recognition ability of the system. Compared with channels that rely only on a single modality, dual-modal fusion shows higher accuracy and stronger robustness in gesture recognition and material recognition tasks. The fused system can complement the signal interference that may occur in the single-modal channel in complex environments and improve the ability to resist noise; in the feature space, the joint representation of static and dynamic information provides the system with richer spatiotemporal features, which helps to accelerate training convergence and reduce the risk of overfitting. Therefore, dual-modal is not only superior to single-modal channels in recognition accuracy, but also shows obvious advantages in system stability and generalization ability.

**Note S8. Explanation of the advantages of the 1D-CNN algorithm.**

In the system prototype stage, the simplicity and real-time performance of the algorithm are prioritized to verify the hardware performance and multimodal signal fusion advantages of the proposed BDR e-skin. The specific reasons are as follows:

(1) Real-time requirements: 1D-CNN has a natural advantage in time series signal processing and can quickly output classification results after a single touch. Compared with two-dimensional convolutional or recurrent neural networks, its inference latency is extremely low, which can fully meet the strict requirements of human-computer interaction and robot feedback for “unperceived” response speed.

(2) Computing resource limitations: On embedded MCUs or resource-constrained control boards, the 1D-CNN structure is lightweight and has few parameters. It not only avoids the long-term dependency and gradient vanishing problems, but also significantly reduces storage and computing overhead, making it easier to promote and deploy on wearable or soft robot platforms in the future.

**Note S9. Explanation of** **high material cognition accuracy under different pressures.**

To effectively reduce the interference introduced by contact pressure fluctuations and improve the robustness and generalization ability of the material identification system, a multi-level strategy has been adopted at the beginning of system design and algorithm construction, as follows:

(i) Signal standardization and normalization preprocessing: During the signal acquisition phase, all triboelectric signals are first normalized to eliminate the differences in amplitude of the original signals, allowing the model to focus more on the waveform and frequency domain characteristics of the signals. This preprocessing is automatically completed within the embedded algorithm on the main control end, ensuring that the original data is at a uniform scale before entering the classification network, thereby effectively suppressing output drift caused by different contact pressures.

(ii) Introduction of multi-dimensional feature modeling and learning mechanism: The constructed 1D-CNN model not only inputs the time series of the triboelectric voltage signal, but also simultaneously introduces its spectrum information (such as main frequency distribution, waveform envelope, peak density, etc.) and attenuation characteristics, so that the model can capture the “intrinsic characteristics” of the material triboelectric behavior from a higher dimension, weakening the dependence on instantaneous pressure value changes. This feature learning mechanism enables the triboelectric behavior of the same material to be stably identified even under a certain pressure disturbance.

(iii) Perturbation enhancement strategy in dataset construction: During the model training phase, we systematically introduced data under different contact pressure ranges and constructed a training sample set containing 500 sets of different contact pressures. This perturbation-enhanced dataset effectively improves the model’s adaptability and generalization performance to external perturbations, thereby achieving high-accuracy identification under non-ideal conditions.

**Supplementary Figure**


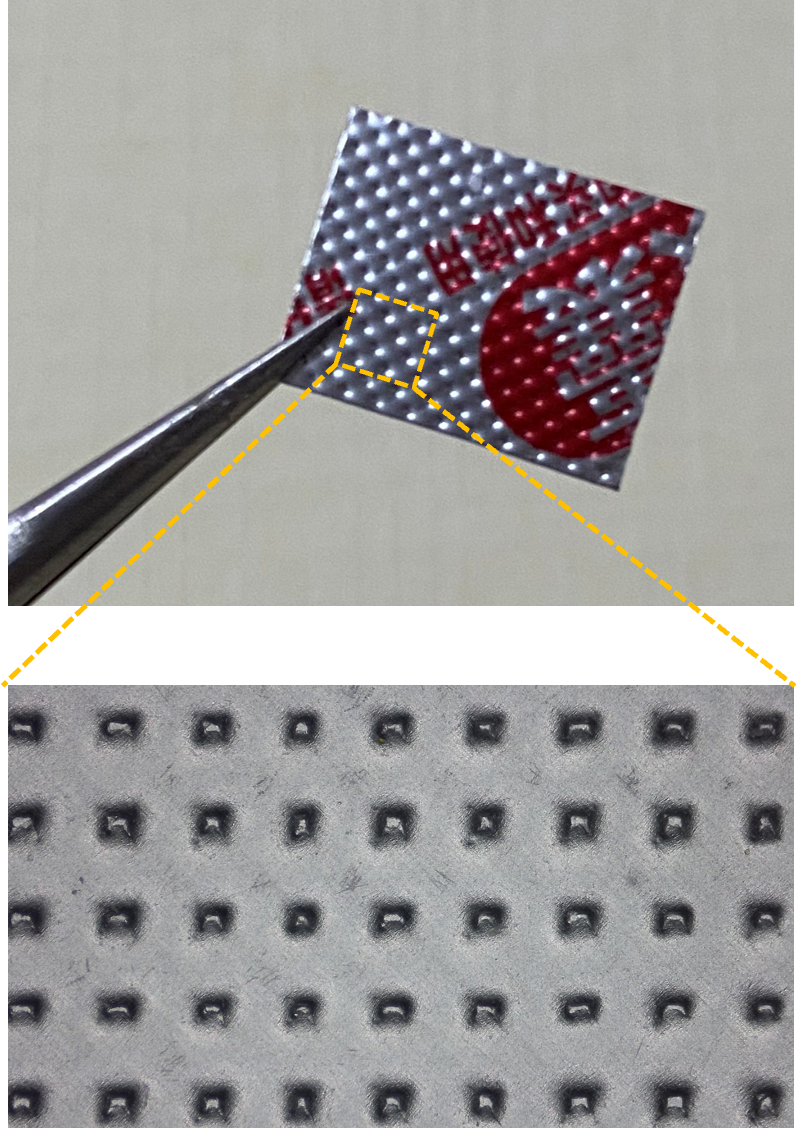


**Figure S1.** Photograph and micrograph of the commercial tablet packaging board.


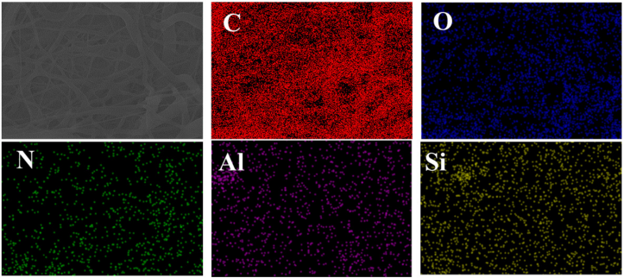


**Figure S2.** EDS elemental characterization of TPU/Sericite fiber membrane.


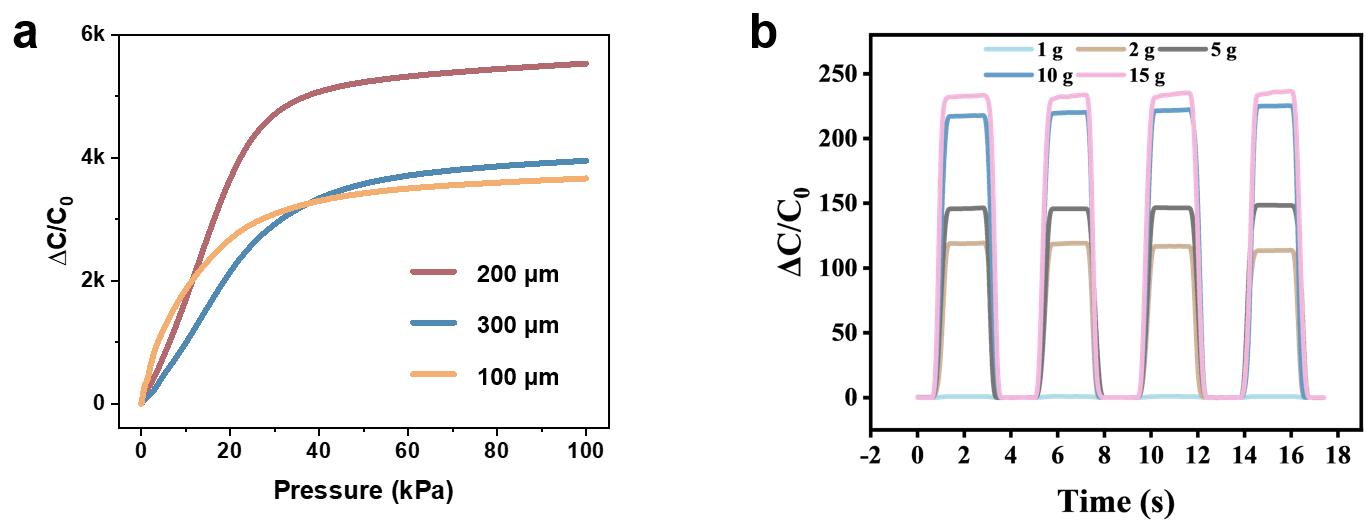


**Figure S3.** a) Capacitive response of in iontronic units with different spacer thicknesses under different pressures. b) Repetitive testing at different pressures.


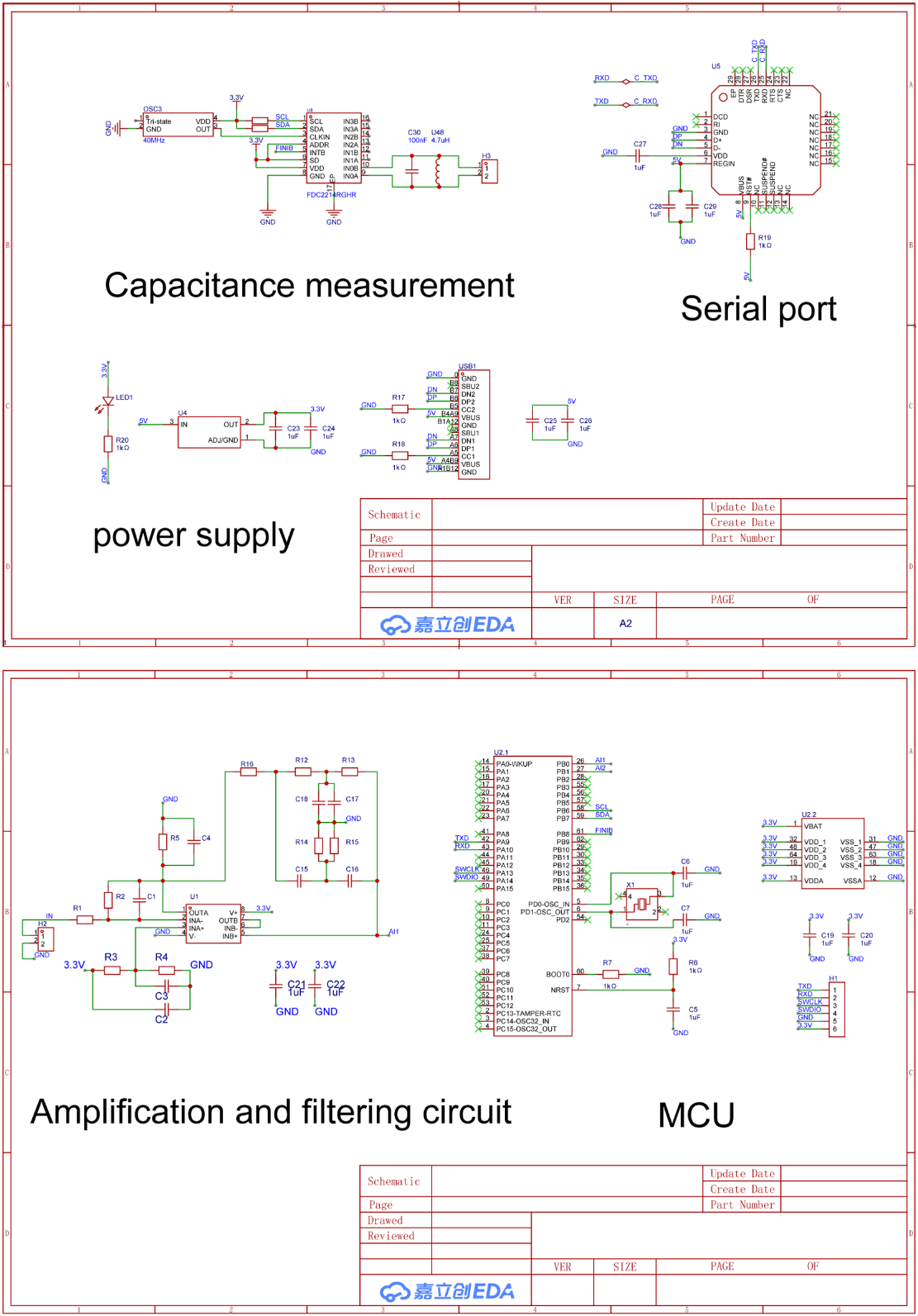


**Figure S4.** Circuit diagram of the constructed hardware circuit.


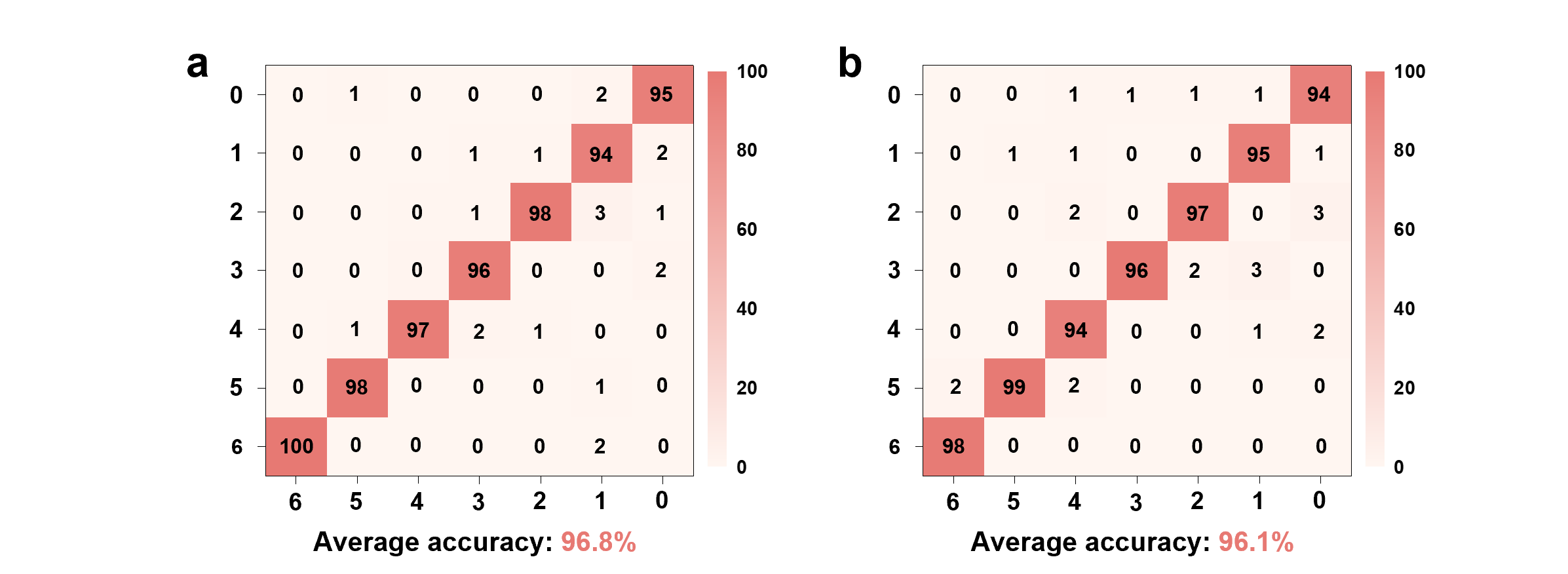


**Figure S5.** a) Confusion matrix showing classification accuracy (%) for sign language gesture cognition based on capacitance signals. b) Confusion matrix showing classification accuracy (%) for sign language gesture cognition based on voltage signals.

**
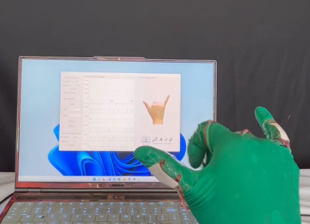
**

**Figure S6.** Real-time display interface for different digital gesture cognition.

**
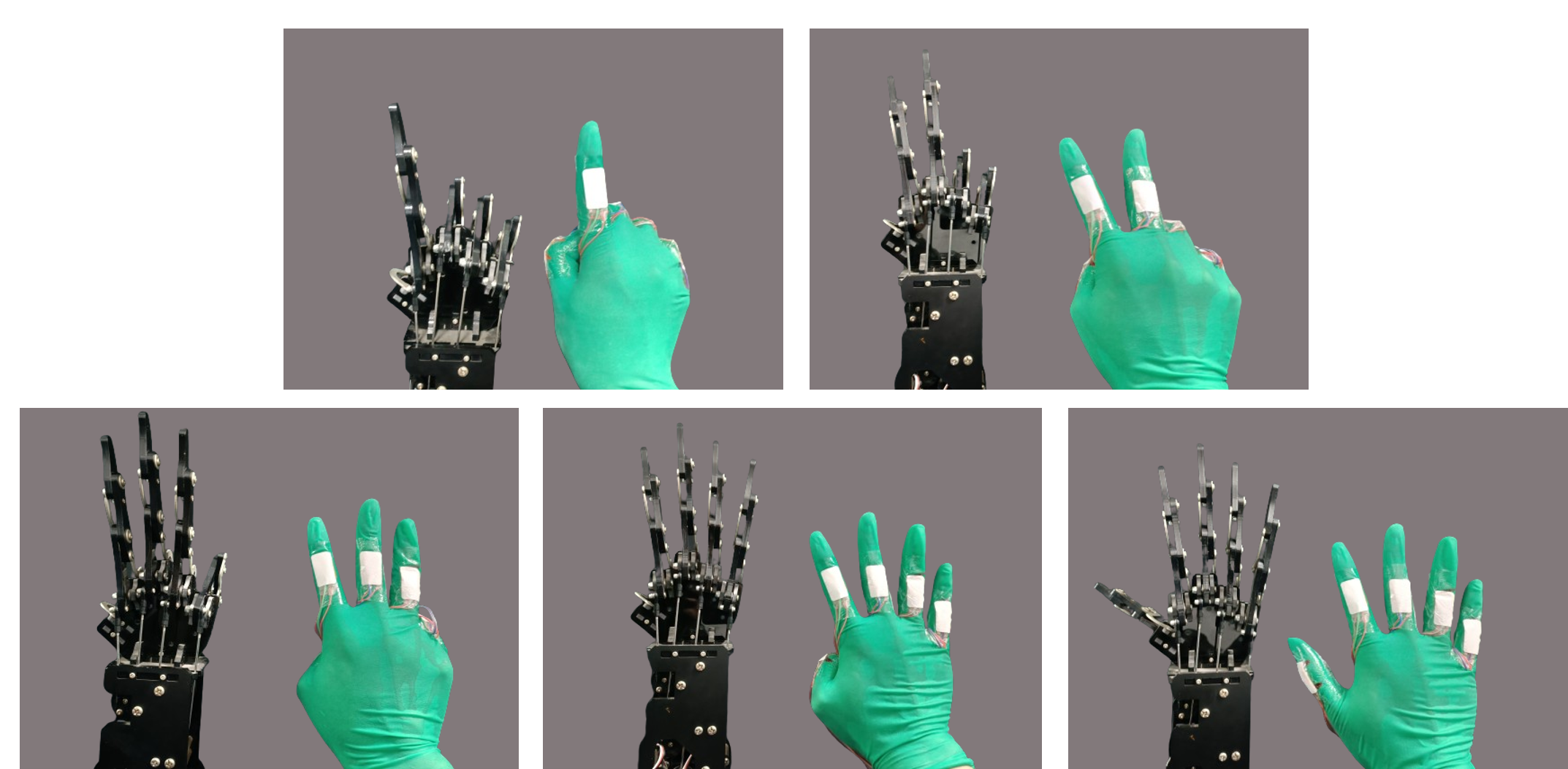
**

**Figure S7.** Real-time interaction between intelligent glove and robot hand.


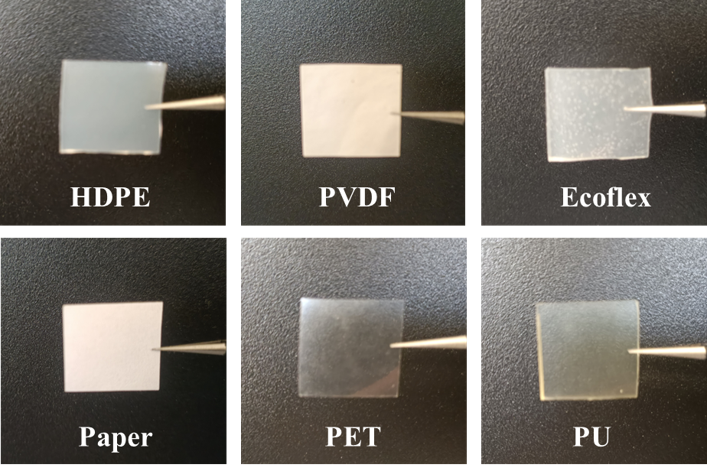


**Figure S8.** Photos of the selected 6 samples.


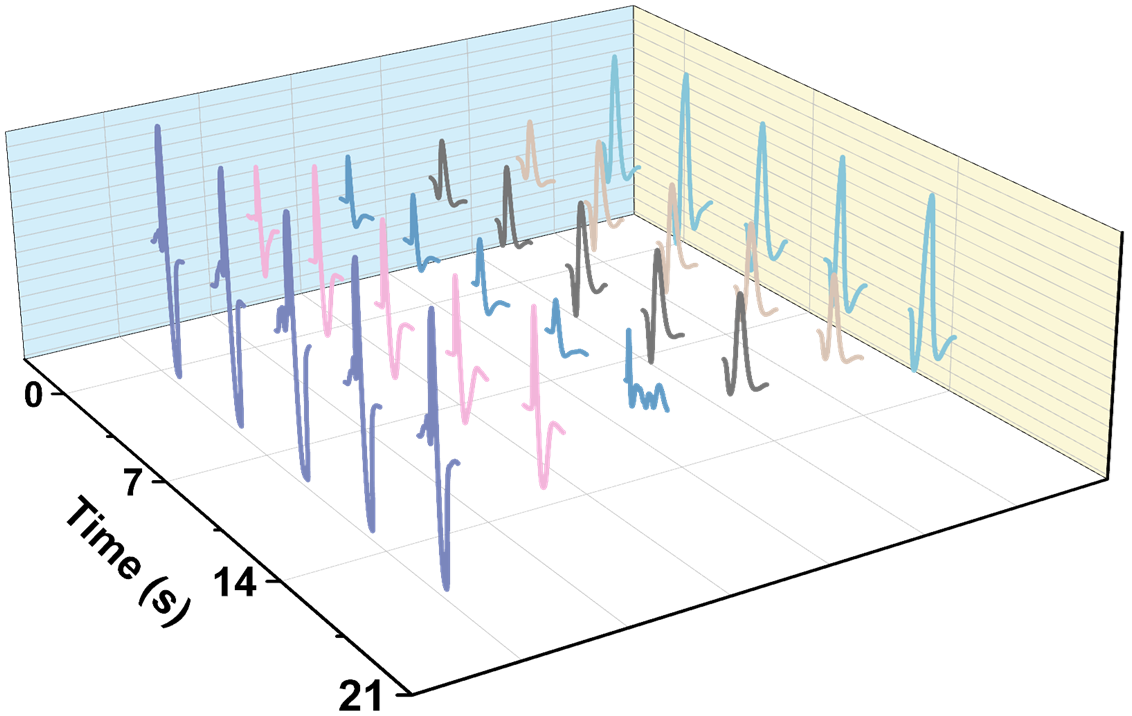


**Figure S9.** Extracted voltage signal waveforms when in contact with different materials.


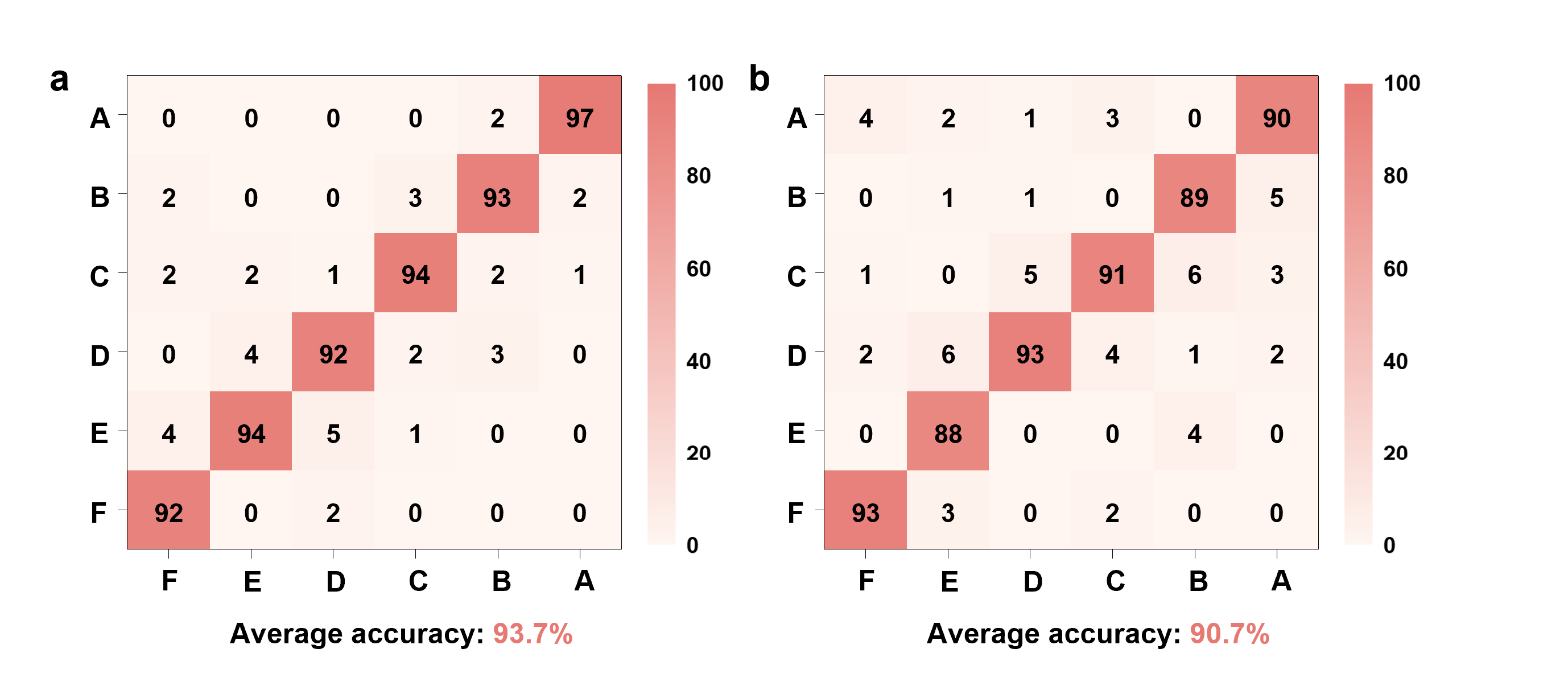


**Figure S10.** a) Confusion matrix showing classification accuracy (%) for autonomous material cognition based on capacitance signals. b) Confusion matrix showing classification accuracy (%) for autonomous material cognition based on voltage signals.


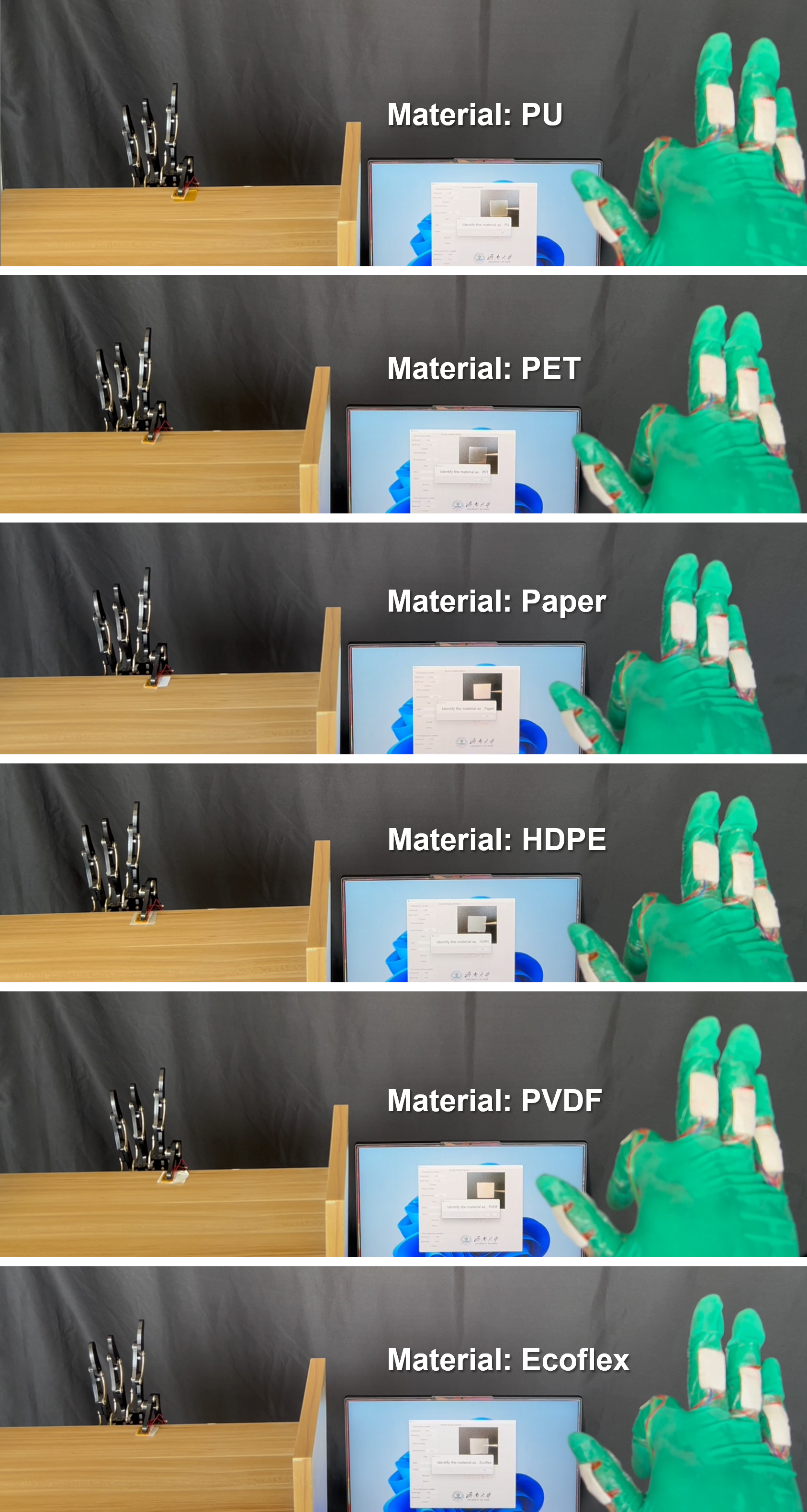


**Figure S11.** Overview of the application scenario of the constructed intelligent autonomous material cognition system.

**Supplementary Table**

**Table S1. Basic physical properties of the materials used.**

| Material | Key parameters | Value range |
| --- | --- | --- |
| AM/agarose/NaCl ionic hydrogel | Ionic conductivity | ~1.2×10^-3^ S/cm |
|  | Tensile strain range | ~150% |
|  | Quality retention (evaporation stability) | >85% (48 h) |
| Ag electrode | Conductivity | ~6.3×10^7^ S/m |
|  | Contact resistance | <10 Ω |
| TPU/sericite fiber membrane | Tensile strength | ~1.5-2.5 MPa |
|  | Young’s modulus | ~3-6 MPa |
|  | Average fiber diameter | ~400-600 nm |
|  | Triboelectric performance stability | >3000 cycles without noticeable attenuation |

**Table S2.** **Comprehensive sensing performance comparison of recently high-level work (iontronic unit).**

| **Ref.** | **Sensitivity**  **[kPa^-1^]** | **Response [ms]** | **Recovery [ms]** | **LOD [Pa]** |
| --- | --- | --- | --- | --- |
| [4] | 62 | 15 | 25 | 0.1 |
| [5] | 3.45 | 28 | 39.2 | 0.21 |
| [6] | 4.2 | 150 | - | 0.5 |
| [7] | 132.02 | 20 | 20 | 0.0133 |
| [8] | 19 | 48 | 64 | 0.05 |
| [9] | 21.67 | 60 | 30 | 3.4 |
| [10] | 4.5 | 50 | 50 | 0.2 |
| [11] | 3.997 | 120 | 60 | 4.7 |
| [12] | 59.15 | 22.4 | 28 | - |
| [13] | 4.8 | 16.8 | 16.8 | 5 |
| **This work** | **172** | **11.2** | **11.2** | **0.5** |

**Table S3. Detailed parameters of each layer of the 1D-CNN model.**

| **Network layer** | **Input** | **Output** |
| --- | --- | --- |
| Conv1d(1, 32, 6) | (1, 4200) | (32, 4195) |
| BatchNorm1d(32)+ ReLU() | (32, 4195) | (32, 4195) |
| AvgPool1d(5) | (32, 4195) | (32, 839) |
| Conv1d(32, 32, 5) | (32, 839) | (32, 835) |
| BatchNorm1d(32)+ ReLU() | (32, 835) | (32, 835) |
| AvgPool1d(5) | (32, 835) | (32, 167) |
| Conv1d(32, 16, 8) | (32, 167) | (16, 160) |
| BatchNorm1d(16)+ ReLU() | (16, 160) | (16, 160) |
| AvgPool1d(5) | (16, 160) | (16, 32) |
| Flatten() | (16, 32) | (512) |
| Linear(512, 64) | (512) | (64) |
| Sigmoid() | (64) | (64) |
| Linear(64, 6) | (64) | (6) |
| Sigmoid() | (6) | (6) |

**Table S4. Elastic modulus of 6 materials**

| **Materials** | **Elastic modulus** |
| --- | --- |
| Ecoflex | 100-200 kPa |
| PU | 1-10 MPa |
| HDPE | 1 GPa |
| Paper | 1.9 GPa |
| PVDF | 2-3 GPa |
| PET | 2.5-4 GPa |

**Table S5. Comparison of related literature based on other material identification in terms of identification dimensions and system capabilities.**

| **Comparison dimensions** | **This work** | **[14] AFM (2024)** | **[15] Small (2023)** | **[16] Small (2024)** | **[17] SA (2020)** |
| --- | --- | --- | --- | --- | --- |
| Perception mechanism | Bionic SA-FA dual-mode perception (static + dynamic), simulating human skin dual receptors | Janus structure TENG, enhanced triboelectric output, single-channel dynamic perception | Capacitive + triboelectric dual mode, supports contactless perception | Thermoelectric dual network hydrogel, based on thermoelectric mechanism | Piezoresistive + thermoelectric + triboelectric three-effect synergy, basic multimodal perception |
| Identification dimensions | Three-dimensional recognition: material type + electronegativity + softness and hardness | Identify material types based on triboelectric signals + deep learning | Only identifies a single metal/polymer/ skin type, limited dimension | Identifiable material types, relying on thermal property differences and machine learning | Table lookup method to identify 10 materials |
| System capabilities | Build a closed-loop system of perception-judgment-execution, integrating gloves and feedback control | Signal acquisition and classification only through linear motors, not yet integrated into robot hands | Interface interaction is the main function, no cognition/ judgment function | Machine learning assisted classification, no execution feedback mechanism | Data collection + table lookup, no closed-loop control |
| Performance | Sensitivity 172 kPa^-1^, response time 11.2 ms, accuracy 96.2% | High output voltage, lack of recognition speed/accuracy data | Demonstrates interactive capabilities, no recognition accuracy reported | Thermal response lag, accuracy of about 95% | Sensitivity 15.22 kPa^-1^, response time <74 ms |
| Overall advantages | Achieve a human-like tactile cognition closed-loop system, leading in functions + integration + application | Focus on TENG performance, no robot cognition and control integration | Interface friendly but shallow cognition, limited dimension | Perceive new ideas but lack of real-time and systematicity | General integration, no intelligent cognitive chain built |

**Table S6. Experimental parameters of Ag deposition on TPU/sericite fiber membrane by a direct current Magnetron sputtering.**

| Ar flow (sccm) | 30 |
| --- | --- |
| direct current power (W) | 70 |
| Deposition pressure (Pa) | 0.5 |
| Deposition time (min) | 30 |

**Supplementary Movie**

**Movie S1.** Real-time display interface for different digital gesture cognition.

**Movie S2.** Real-time interaction between intelligent glove and robot hand.

**Movie S3.** Intelligent autonomous material cognition system for cognition of material species.

**References**

[1] S. R. A. Ruth, V. R. Feig, H. Tran, Z. Bao, *Adv. Mater.* **2020**, 30, 2003491.

[2] H. Niu, H. Zhang, W. Yue, S. Gao, H. Kan, C. Zhang, C. Zhang, J. Pang, Z. Lou, L. Wang, Y. Li, H. Liu, G. Shen, *Small* **2021**, 17, 2100804.

[3] X. Zhao, S. Zhao, X. Zhang, Z. Su, *Nanoscale* **2023**, 15, 5111.

[4] G.-Y. Gou, X.-S. Li, J.-M. Jian, H. Tian, F. Wu, J. Ren, X.-S. Geng, J.-D. Xu, Y.-C. Qiao, Z.-Y. Yan, G. Dun, C. W. Ahn, Y. Yang, T.-L. Ren, *Sci. Adv.* **2022**, 8, eabn2156.

[5] W. Xiong, F. Zhang, S. Qu, L. Yin, K. Li, Y. Huang, *Nat. Commun.* **2024**, 15, 5596.

[6] S. W. Kim, J.-H. Lee, H. J. Ko, S. Lee, G. Y. Bae, D. Kim, G. Lee, S. G. Lee, K. Cho, *ACS Nano* **2024**, 18, 3151.

[7] X. Xu, B. Yan, *Adv. Mater.* **2023**, 35, 2303410.

[8] J.-H. Zhang, Z. Li, J. Xu, J. Li, K. Yan, W. Cheng, M. Xin, T. Zhu, J. Du, S. Chen, X. An, Z. Zhou, L. Cheng, S. Ying, J. Zhang, X. Gao, Q. Zhang, X. Jia, Y. Shi, L. Pan, *Nat. Commun.* **2022**, 13, 5839.

[9] S. Wang, Y. Yao, W. Deng, X. Chu, T. Yang, G. Tian, Y. Ao, Y. Sun, B. Lan, X. Ren, X. Li, T. Xu, L. Huang, Y. Liu, J. Lu, W. Yang, *ACS Nano* **2024**, 18, 11183.

[10] Q. Su, Q. Zou, Y. Li, Y. Chen, S.-Y. Teng, J. T. Kelleher, R. Nith, P. Cheng, N. Li, W. Liu, S. Dai, Y. Liu, A. Mazursky, J. Xu, L. Jin, P. Lopes, S. Wang, Sci. Adv. **2021**, 7, eabi4563.

[11] X. Wang, G. Wu, X. Zhang, F. Lv, Z. Yang, X. Nan, Z. Zhang, C. Xue, H. Cheng, L. Gao, *Adv. Mater.* **2025**, 37, 2410312.

[12] X. Wang, H. Niu, S. Gao, G. Shen, Y. Li, *Nano Lett.* **2025**, 25, 5784.

[13] J. Gao, H. Niu, Y. Li, Y. Li, *Adv. Funct. Mater.* **2025**, 35, 2418463.

[14] C. Jin, C. Zhang, P. Yan, M. Jiang, R. Yin, K. Li, W. Zhao, Z. Bai, *Adv. Funct. Mater.* **2024**, 34, 2402233.

[15] H. L. Wang, T. Chen, B. Zhang, G. Wang, X. Yang, K. Wu, Y. Wang, *Small* **2023**, 19, 2206830.

[16] Y. Li, W. Wang, X. Cui, N. Li, X. Ma, Z. Wang, Y. Nie, Z. Huang, H. Zhang, *Small* **2024**, 21, 2405911.

[17] Y. Wang, H. Wu, L. Xu, H. Zhang, Y. Yang, Z. L. Wang, *Sci. Adv.* **2020**, 6, eabb9083.
